# OnabotulinumtoxinA (botulinum toxin type A) for the treatment of Japanese patients with overactive bladder and urinary incontinence: Results of single‐dose treatment from a phase III, randomized, double‐blind, placebo‐controlled trial (interim analysis)

**DOI:** 10.1111/iju.14176

**Published:** 2020-01-20

**Authors:** Osamu Yokoyama, Masashi Honda, Tomonori Yamanishi, Yuki Sekiguchi, Kenji Fujii, Takashi Nakayama, Takao Mogi, Takeya Kitta, Chikara Ohyama, Koichi Kambe, Yoshikatsu Tanahashi, Isoji Sasagawa, Mikinobu Ootani, Eiyu Nozawa, Toshihiko Saito, Fumio Manabe, Yasutomo Suzuki, Hikaru Tomoe, Satoru Takahashi, Nozomu Kawata, Hisashi Matsushima, Taketo Kawai, Manami Kinjo, Takashi Fukagai, Atsuko Takahashi, Fumio Nakajima, Haruaki Sasaki, Norihiko Okuno, Toshiyuki Itoi, Yoshiyuki Ishiura, Atsushi Mizokami, Yosuke Matsuta, Norifumi Sawada, Kosei Miwa, Atsushi Otsuka, Yasuhito Funahashi, Masaki Yoshida, Motoi Tobiume, Akihiro Kawauchi, Hiroshi Okuno, Terukazu Nakamura, Noriko Ninomiya, Chie Matsushita, Atsushi Takenaka, Takeshi Watanabe, Toyohiko Watanabe, Noritaka Ishito, Yasuyo Yamamoto, Hiroshi Ikeda, Narihito Seki, Shigehiro Kanegae, Kenichi Suyama, Kazuyuki Sagiyama, Noriaki Tokuda, Hideki Sakai, Hiroaki Nishimura, Motoshi Kawahara

**Affiliations:** ^1^ Department of Urology Faculty of Medical Science University of Fukui Fukui Japan; ^2^ Department of Urology Tottori University Faculty of Medicine Tottori Japan; ^3^ Department of Urology Dokkyo Medical University Tochigi Japan; ^4^ Yokohama Motomachi Women’s Clinic LUNA Kanagawa Japan; ^5^ Medicines Development Japan Development Division GlaxoSmithKline Tokyo Japan; ^6^ Biomedical Data Sciences Department Japan Development Division GlaxoSmithKline Tokyo Japan

**Keywords:** botulinum toxin type A, onabotulinumtoxinA, overactive bladder, randomized controlled trial, urinary incontinence

## Abstract

**Objective:**

To evaluate the efficacy and safety of onabotulinumtoxinA (botulinum toxin type A) 100 U in patients with overactive bladder and urinary incontinence.

**Methods:**

This was a phase III, randomized, double‐blind, placebo‐controlled trial in Japanese patients who were inadequately managed with overactive bladder medications (anticholinergics and/or β_3_‐adrenergic receptor agonists). Eligible patients were randomized 1:1 to receive a single dose of either onabotulinumtoxinA or placebo into the detrusor muscle (*n* = 124 each). The primary end‐point was the change in the number of daily urinary incontinence episodes at week 12 from baseline. Secondary end‐points included volume voided per micturition, other symptomatic measures (urinary urgency incontinence, micturition, urgency and nocturia) and patient‐reported outcomes.

**Results:**

In the onabotulinumtoxinA group, there was a significantly greater decrease from baseline in the mean number of daily urinary incontinence episodes compared with the placebo group (2.16; *P* < 0.001), and significantly greater improvement for all secondary end‐points (*P* < 0.05). Urinary tract infection, dysuria, urinary retention and post‐void residual urine volume increased represented adverse events occurring at a higher rate in the onabotulinumtoxinA group. The majority of these were mild or moderate in severity.

**Conclusions:**

Statistically significant and clinically relevant improvements in symptoms and patient‐reported outcomes, and tolerability were seen in patients with overactive bladder and urinary incontinence who had been inadequately managed with overactive bladder medications after using onabotulinumtoxinA.

**Abbreviations & Acronyms**
AE: adverse event
BoNTA: onabotulinumtoxinA
CIC: clean intermittent catheterization
FAS: full analysis set
KHQ: King’s Health Questionnaire
MMRM: mixed model for repeated measures
OAB: overactive bladder
OABSS: overactive bladder symptom score
PRO: patient‐reported outcome
PVR: post‐void residual
SAE: serious adverse event
SD: standard deviation
SE: standard error
TBS: treatment benefit scale
TRPV1: transient receptor potential vanilloid 1
UI: urinary incontinence
UTI: urinary tract infection
UUI: urinary urgency incontinence

## Introduction

OAB is a prevalent and chronic disabling condition, defined by the storage symptoms of “urgency, with or without urge incontinence, usually with frequency and nocturia” by the International Continence Society.^1^ These symptoms significantly decrease quality of life, affecting daily activities, such as physical and social activity, and have a major impact on mental health.^2^, ^3^, ^4^ In addition, OAB is considered to be a disease that poses a significant economic burden, due not only to the treatment of the symptoms, but also to lost working days.^5^ An epidemiological survey in Japan showed that the prevalence of OAB in people aged ≥40 years is 12.4%, and that it increases with advanced age, affecting an estimated 10.4 million people in Japan.^6^, ^7^ Considering that the population of Japan is aging, establishing appropriate treatment regimens for OAB is considered to be an increasingly important issue.

Pharmacotherapy, usually using anticholinergics or β_3_‐adrenergic receptor agonists, is the mainstay of treatment for OAB.^6^ However, these drugs often have poor efficacy and/or tolerability, leading to low adherence and their discontinuation.^8^, ^9^ Currently, there are few acceptable alternative treatment options for patients who cannot be adequately managed with the available pharmacotherapies. These include magnetic stimulation and sacral nerve stimulation in Japan.^6^ However, these two options are not always suitable for the patients as, respectively, they necessitate frequent hospital visits for use of the device, and repeated operations to replace the neurostimulator due to battery exhaustion.^10^, ^11^

Botulinum toxin type A is produced from *Clostridium botulinum* and inhibits neurotransmitter release from presynaptic nerve terminals, such as motor nerves and parasympathetic nerves, producing effects that include muscle relaxation. Botulinum toxin type A administered into the detrusor muscle of the bladder inhibits acetylcholine release, suppressing the involuntary contraction of the muscle.^12^ In addition, it has been suggested that botulinum toxin type A might influence an afferent pathway, suppressing urgency by blocking release of neurotransmitters, such as substance P and adenosine triphosphate, and inhibiting the expression of P2X_3_ receptor and TRPV1.^13^, ^14^ At present, some therapeutic products of botulinum toxin type A are available worldwide, such as BoNTA (BOTOX; Allergan, Dublin, Ireland) and abobotulinumtoxinA (Dysport).

The efficacy and safety of BoNTA 100 U for OAB were established in two large, phase III placebo‐controlled trials and a long‐term extension study carried out in North America and Europe.^15^, ^16^, ^17^ Currently, BoNTA is approved and recommended in a number of countries as an alternative therapy for OAB patients in whom prior therapies, such as anticholinergics and/or β_3_‐adrenergic receptor agonists, have failed.^18^, ^19^ In Japan, BoNTA has not been approved for OAB, and the use of BoNTA has been reported in only one small sample‐sized open‐label study and in some case reports.^20^, ^21^

Thus, in the present phase III, randomized, double‐blind, placebo‐controlled trial, we evaluated the efficacy and safety of BoNTA 100 U in Japanese OAB and UI patients who were not adequately managed with anticholinergics and/or β_3_‐adrenergic receptor agonists.

## Methods

### Study

#### Participants

Patients aged ≥20 years with OAB who experienced three or more episodes of UUI and a mean of eight or more micturitions per day in a 3‐day diary were enrolled in the study. All eligible patients had not been adequately managed with one or more anticholinergics and/or β_3_‐adrenergic receptor agonists for OAB treatment, due to insufficient efficacy or intolerable side‐effects. Patients were not permitted to use these other OAB medications from 7 days before screening initiation until the end of the study. In addition, the patients had to be willing to use CIC if required by the investigators. Exclusion criteria included previous botulinum toxin treatment for any urological condition; stress‐dominant mixed UI; any diseases, functional abnormalities or bladder surgery that might affect bladder function; and a PVR urine volume >100 mL. Patients who met the eligibility criteria were randomized to either BoNTA 100 U or a placebo at a ratio of 1:1, and were stratified according to UUI episodes (≤9 or ≥10 episodes) in the 3‐day diary during the screening period.

#### Design

**Figure 1:**
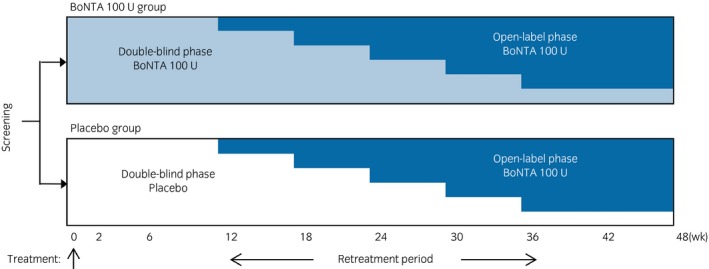
Study design.

This was a phase III, randomized, double‐blind, placebo‐controlled trial carried out from August 2016 to November 2018 at 53 sites in Japan (ClinicalTrials.gov NCT02820844) in compliance with Good Clinical Practice regulations. The study was approved by the institutional review board of each study site. This study consisted of a screening period (within 4 weeks) and a treatment period with two phases: (i) the double‐blind phase (randomized, placebo‐controlled design) to evaluate the efficacy and safety of a single dose of BoNTA 100 U compared with a placebo; and (ii) the open‐label phase, after the double‐blind phase, to evaluate the efficacy and safety of repeated doses of BoNTA 100 U. The total treatment period, including both phases, was 48 weeks, as shown in Figure 1. Here, we report the efficacy and safety results of the double‐blind phase up to week 12, based on the interim analysis data for all patients up to 24 weeks after the first treatment.

#### Treatment

Administration of antibiotic agents was initiated 1–3 days before injection of the study drug, and was continued for 1–3 days post‐injection for prophylaxis of UTI. Instillation of local anesthesia to the bladder and/or sedation could be carried out at the discretion of the investigator. BoNTA 100 U (BOTOX; Allergan) or a placebo, reconstituted with 10 mL of saline, was injected and evenly distributed at 20 sites in the detrusor muscle using a rigid or flexible cystoscope and a 22‐G injection needle (BoNee; Coloplast, Humlebaek, Denmark), avoiding the trigone and dome of the bladder.

### Efficacy and safety evaluations

The primary end‐point was the change from baseline in the number of daily UI episodes at week 12. The major secondary end‐point was the change from baseline in the volume voided per micturition at week 12. Other secondary end‐points were the change from baseline in the number of episodes of daily UUI, micturition, urgency and nocturia. To collect the data for these end‐points, the 3‐day diary was completed during screening and in the week before each post‐treatment visit. PRO was assessed using KHQ,^22^ OABSS^23^ and TBS.^24^

The number of AEs, PVR urine volume, the use of CIC for urinary retention/elevated PVR urine volume and clinical laboratory tests (hematology, chemistry and urinalysis including urine culture) were used to assess safety, in addition to the monitoring of vital signs, kidney and bladder ultrasounds, and 12‐lead electrocardiograms. The severity of AEs was assessed using the following categories: (i) mild: an event that is easily tolerated by the participant, causing minimal discomfort and not interfering with everyday activities; (ii) moderate: an event that causes sufficiently discomfort to interfere with normal everyday activities; and (iii) severe: an event that prevents normal everyday activities.

### Statistical analysis

The primary analyses were based on the FAS population (all randomized patients who had at least one post‐baseline efficacy assessment). Safety analyses were based on the patients who had received the single dose of study drug (safety population). A sample size of 240 participants was expected to provide at least 89% joint power at a significance level of 5% to detect both treatment differences for change from baseline in the mean number of daily UI episodes, and the mean volume voided per micturition, simultaneously. This power was calculated from the power for each end‐point (98% for UI, and 91% for volume voided per micturition) and Bonferroni inequality, when it was assumed that the means/SD of the treatment differences were −1.79/3.5 episodes and 30/70 mL, respectively.

The primary end‐point and the major secondary end‐point were analyzed using a MMRM with treatment, site, visit, treatment‐by‐visit interaction, baseline value (baseline UUI episodes of ≤9 or ≥10 episodes were also added for the major secondary end‐point) and baseline‐by‐visit interaction as fixed effects. The data missing after study withdrawal were not imputed for MMRM analysis. As two statistical hypotheses (i.e. the primary and major secondary end‐point analyses) needed to be confirmed simultaneously in this study, multiplicity was not taken into account for these analyses.

## Results

### Patient demographics and baseline disease characteristics

**Figure 2:**
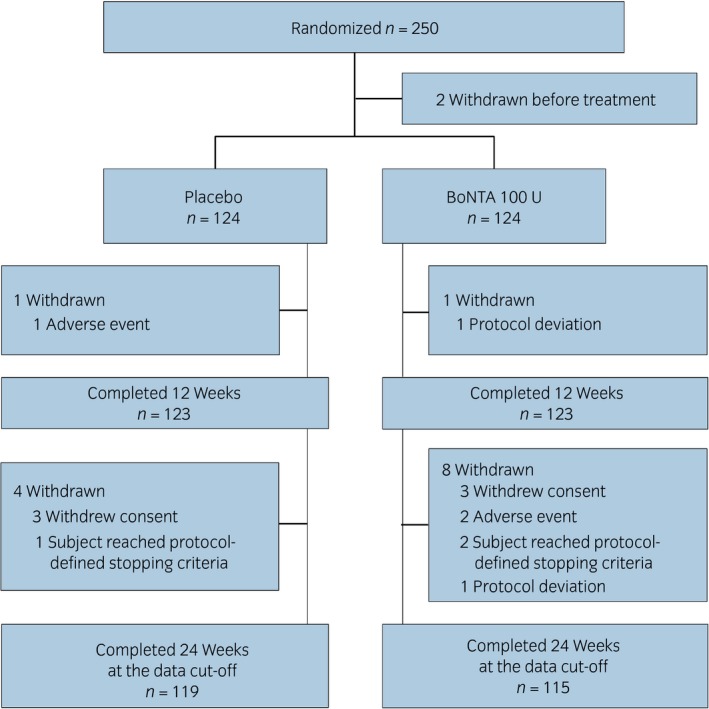
Disposition of the FAS patient population.

A total of 250 patients were randomized into the study. Of these, 248 patients received either BoNTA 100 U (*n* = 124) or a placebo (*n* = 124). Overall, 14 patients discontinued early, two before the primary time point at week 12 (BoNTA, *n* = 1; placebo, *n* = 1), and a further 12 before the data cut‐off at week 24 (BoNTA, *n* = 8; placebo, *n* = 4; Fig. 2). The baseline demographics and disease characteristics were comparable between the groups (Table 1). Lack of efficacy of prior OAB medication was the primary reason for the inadequate management of OAB in both treatment groups.

**Table 1:** Baseline demographics and disease characteristics (FAS population)

	Placebo *n* = 124	BoNTA 100 U *n* = 124
Age (years)	66.2 ± 12.19	65.6 ± 12.43
Female (*n*/%)	94/76	92/74
Weight (kg)	59.20 ± 12.432	58.27 ± 10.778
Height (cm)	156.46 ± 7.477	156.00 ± 8.171
OAB history (years)	4.19 ± 4.041	5.22 ± 4.842
PVR urine volume (mL)	18.10 ± 24.687	19.15 ± 24.671
No. daily episodes		
UI	6.12 ± 3.866	7.01 ± 4.782
UUI	5.71 ± 3.535	6.56 ± 4.722
Micturition	12.72 ± 3.333	12.20 ± 3.712
Urgency	9.54 ± 4.175	9.18 ± 4.780
Nocturia	1.86 ± 1.412	1.71 ± 1.476
Volume voided per micturition (mL)	130.56 ± 48.820	132.06 ± 50.831
KHQ domain score		
Role limitations	60.84 ± 27.410	62.77 ± 29.071
Social limitations	46.57 ± 28.475	46.64 ± 30.510
OABSS total score	11.7 ± 2.11	11.5 ± 1.89

All data except for Female (*n*/%) are expressed as mean ± SD.

### Efficacy

#### Primary end‐point

**Figure 3:**
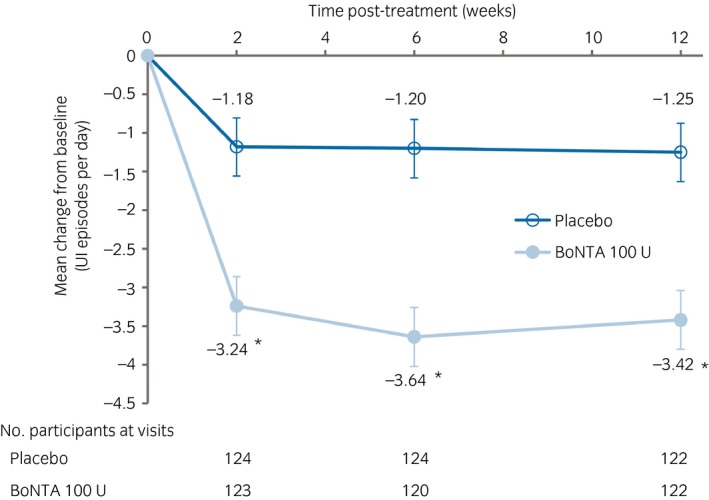
Change from baseline in the mean number of daily UI episodes. Values: adjusted mean; error bars: SE. *P < 0.001 versus placebo. The data were analyzed using a MMRM with treatment, site, visit, treatment‐by‐visit interaction, baseline value and baseline‐by‐visit interaction as fixed effects.

At week 12, there was a greater decrease from baseline in the mean number of daily UI episodes in the BoNTA group than in the placebo group (Fig. 3; Table 2), with a statistically significant between‐group difference of −2.16 (95% CI −3.14, −1.18; *P* < 0.001). A significant between‐group difference was sustained from the first post‐treatment evaluation at week 2 until the evaluation at week 12 (*P* < 0.001 for each time point).

**Table 2:** Change from baseline in the efficacy measures evaluated by the diary up to post‐treatment week 12 (FAS population)

	Adjusted mean change from baseline ± SE		Difference *vs* placebo (95% CI)	*P*‐value
	Placebo, *n* = 124	BoNTA 100 U, *n* = 124		
No. daily UI episodes†				
Week 2	−1.18 ± 0.372	−3.24 ± 0.379	−2.06 (−3.03, −1.09)	<0.001
Week 6	−1.20 ± 0.374	−3.64 ± 0.382	−2.44 (−3.41, −1.46)	<0.001
Week 12	−1.25 ± 0.375	−3.42 ± 0.381	−2.16 (−3.14, −1.18)	<0.001
No. daily UUI episodes†				
Week 2	−0.99 ± 0.353	−3.17 ± 0.360	−2.18 (−3.10, −1.26)	<0.001
Week 6	−1.02 ± 0.362	−3.46 ± 0.369	−2.44 (−3.39, −1.49)	<0.001
Week 12	−1.02 ± 0.363	−3.13 ± 0.370	−2.12 (−3.07, −1.17)	<0.001
No. daily micturitions‡				
Week 2	−0.44 ± 0.307	−0.58 ± 0.312	−0.14 (−0.94, 0.65)	0.723
Week 6	−0.29 ± 0.297	−1.78 ± 0.303	−1.49 (−2.25, −0.72)	<0.001
Week 12	−0.42 ± 0.306	−1.87 ± 0.311	−1.45 (−2.24, −0.66)	<0.001
No. daily urgency episodes‡				
Week 2	−1.20 ± 0.432	−2.10 ± 0.442	−0.90 (−2.02, 0.21)	0.112
Week 6	−1.47 ± 0.429	−3.32 ± 0.440	−1.85 (−2.96, −0.74)	0.001
Week 12	−1.17 ± 0.419	−3.40 ± 0.429	−2.23 (−3.31, −1.16)	<0.001
No. daily nocturia episodes‡				
Week 2	−0.05 ± 0.111	−0.10 ± 0.113	−0.05 (−0.33, 0.24)	0.751
Week 6	−0.08 ± 0.115	−0.28 ± 0.118	−0.19 (−0.49, 0.11)	0.209
Week 12	0.03 ± 0.125	−0.30 ± 0.127	−0.33 (−0.66, 0.00)	0.048
Volume voided per micturition (mL)‡				
Week 2	5.40 ± 4.105	15.17 ± 4.175	9.78 (−0.77, 20.33)	0.069
Week 6	2.31 ± 4.535	30.26 ± 4.624	27.96 (16.08, 39.84)	<0.001
Week 12	−0.22 ± 4.329	29.47 ± 4.390	29.69 (18.47, 40.91)	<0.001

† MMRM with treatment, site, visit, treatment‐by‐visit interaction, baseline value and baseline‐by‐visit interaction as fixed effects.

‡ MMRM with treatment, site, visit, baseline UUI episodes over the 3‐day diary (≤9 or ≥10), treatment‐by‐visit interaction, baseline value and baseline‐by‐visit interaction as fixed effects.

#### Secondary end‐points

**Figure 4:**
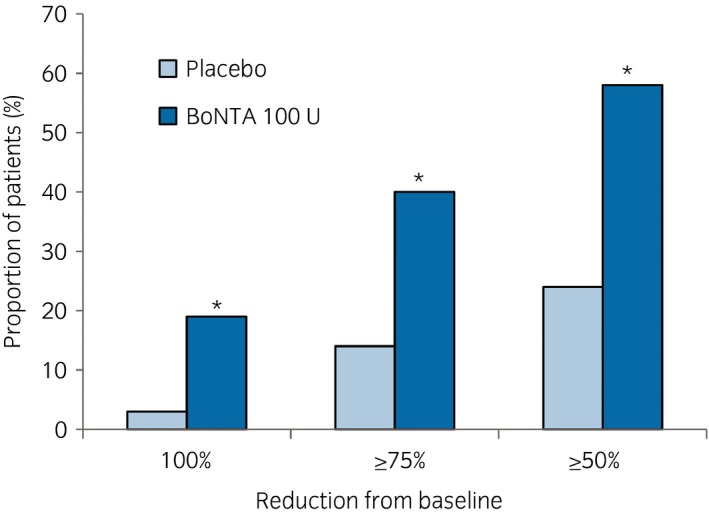
Proportion of patients attaining 100%, ≥75% and ≥50% reduction from baseline in the number of daily UI episodes at week 12. *P < 0.001 versus placebo. The data were analyzed using the Cochran–Mantel–Haenszel test stratified by baseline UUI (≤9 episodes *vs* ≥10 episodes).

There was a significantly greater increase from baseline in the volume voided per micturition at week 12, the major secondary end‐point, in the BoNTA group than in the placebo group, with a between‐group difference of 29.69 mL (95% CI 18.47, 40.91; *P* < 0.001; Table 2). For other secondary end‐points related to OAB symptoms (i.e. the mean numbers of daily UUI episodes, micturition, urgency episodes and nocturia episodes), reductions from baseline at week 12 were also observed in the BoNTA group, and these were statistically significant compared with those of the placebo group (*P* < 0.05). In addition, the proportion of patients “dry” at week 12 (100% reduction in UI from baseline) was significantly higher in the BoNTA group (19%) than in the placebo group (3%; Fig. 4). Improvements in PRO were noted in the KHQ domain scores of “Role Limitations” and “Social Limitations,” and in the OABSS total score, in the BoNTA group in comparison with those of the placebo group. The proportion of patients with a positive response on the TBS at week 12 was significantly (*P* < 0.001) higher in the BoNTA group than in the placebo group (Table 3).

**Table 3:** Summary of PRO results at post‐treatment week 12 (FAS population)

	Placebo *n* = 124	BoNTA 100 U *n* = 124	*P*‐value
Change from baseline in KHQ domain score at week 12 (adjusted mean ± SE)†			
Role limitations	−6.48 ± 2.976	−21.09 ± 2.997	<0.001
Social limitations	−4.95 ± 2.945	−13.36 ± 2.983	0.028
Change from baseline in OABSS total score at week 12 (adjusted mean ± SE)†	−0.7 ± 0.32	−3.4 ± 0.33	<0.001
Number and proportion of patients with a positive treatment response on the TBS up to post‐treatment week 12, *n* (%)‡			
Week 2	27 (22)	75 (60)	<0.001
Week 6	29 (23)	79 (64)	<0.001
Week 12	21 (17)	71 (57)	<0.001

† Analysis of covariance model with treatment, site, baseline UUI episodes over the 3‐day diary (≤9 or ≥10) and baseline value as fixed effects.

‡ Cochran–Mantel–Haenszel test stratified by baseline UUI (≤9 episodes *vs* ≥10 episodes). A positive treatment response was defined as a score of either 1 (greatly improved) or 2 (improved).

### Safety

The AEs that occurred higher within the first 12 weeks in the BoNTA group than in the placebo group, all of which were localized to the urinary tract, were UTI, dysuria, urinary retention and PVR urine volume increased. Incidences of these were 13%, 10%, 6% and 6%, respectively, in the BoNTA group, compared with 7%, 2%, 2% and 0%, respectively, in the placebo group (Table 4). AEs assessed as treatment‐related by the investigator that occurred within the first 12 weeks at a higher incidence in the BoNTA group were also localized to the urinary tract, and included dysuria, PVR urine volume increased, UTI and urinary retention (incidences in the BoNTA group were 8%, 5%, 5% and 5%, respectively). The majority of AEs during this period were mild or moderate in severity.

**Table 4:** Summary of AEs reported for ≥3% of patients in either group during the 12 weeks post‐treatment (safety population)

AE	Placebo, *n* = 124 *n* (%)	BoNTA 100 U, *n* = 124 *n* (%)
All AEs	64 (52)	76 (61)
UTI†	9 (7)	16 (13)
Nasopharyngitis	11 (9)	15 (12)
Dysuria	3 (2)	12 (10)
Urinary retention‡	2 (2)	7 (6)
PVR urine volume increased	0	7 (6)
Cystitis	2 (2)	4 (3)
Hematuria	4 (3)	3 (2)

† UTIs were reported as AEs regardless of the presence of symptoms when the result of urinalysis was positive (bacteriuria with ≥10^5^ colony‐forming unit/mL, and leukocyturia at >5/high‐power field).

‡ Urinary retention was defined when a patient who had an increased PVR urine volume required CIC; PVR urine volume of ≥350 mL (regardless of symptoms) or PVR urine volume ≥200 and <350 mL, and reported associated symptoms (e.g. voiding difficulties or sensation of bladder fullness) for which the investigator deemed CIC to be necessary.

The PVR urine volume was unchanged from baseline in the placebo group, whereas in the BoNTA group it was increased from baseline throughout the 12 weeks. This increase was most pronounced at week 2 (+48.78 mL), diminishing by week 12 (+15.53 mL; Table 5). For most of the patients, post‐treatment PVR urine volume was <100 mL up to week 12. Just 6% of the patients treated with BoNTA showed a PVR urine volume ≥200 mL at week 2, and this decreased to 0% by week 12. The proportion of patients who initiated CIC for urinary retention or elevated PVR during the first 12 weeks was higher in the BoNTA group (6%) than in the placebo group (2%).

**Table 5:** Safety parameters associated with PVR urine volume and CIC initiation up to post‐treatment week 12 (safety population)

	Placebo *n* = 124	BoNTA 100 U *n* = 124
Change from baseline in PVR urine volume up to post‐treatment week 12, mL (adjusted mean ± SE)†		
Week 2	–1.15 ± 6.195	48.78 ± 6.240
Week 6	–1.41 ± 4.730	24.14 ± 4.821
Week 12	1.54 ± 3.830	15.53 ± 3.882
No. and proportion of patients by PVR urine volume category up to post‐treatment week 12, *n* (%)		
Week 2		
<100 mL	120 (97)	93 (76)
≥100 to <200 mL	4 (3)	23 (19)
≥200 to <350 mL	0	6 (5)
≥350 mL	0	1 (<1)
Week 6		
<100 mL	121 (98)	102 (84)
≥100 to <200 mL	3 (2)	19 (16)
≥200 to <350 mL	0	1 (<1)
≥350 mL	0	0
Week 12		
<100 mL	115 (94)	112 (91)
≥100 to <200 mL	7 (6)	11 (9)
≥200 to <350 mL	0	0
≥350 mL	0	0
No. and proportion of patients initiating CIC up to post‐treatment week 12‡, *n* (%)	2 (2)	7 (6)

† Analysis of covariance model with treatment, site, baseline UUI episodes over the 3‐day diary (≤9 or ≥10) and baseline value as fixed effects.

‡ CIC initiation for urinary retention or elevated PVR urine volume.

During the first 12 weeks, only one patient in the placebo group discontinued treatment due to AE (breast cancer). The incidence of SAEs during the first 12 weeks was similar in the BoNTA group (3%) and the placebo group (2%). Of these, dysarthria, which occurred in one patient in the BoNTA group, was assessed as being related to the study drug. There were no clinically relevant differences between the treatment groups with respect to clinical laboratory tests, vital signs, bladder and kidney ultrasounds, or 12‐lead electrocardiograms except in urinalysis, related to the UTI.

## Discussion

This is the first randomized, placebo‐controlled trial in Japanese patients with OAB and UI who were not adequately managed with OAB medications (anticholinergic and/or β_3_‐adrenergic receptor agonist). At week 12 in the double‐blind phase, there were greater improvements in all primary and secondary end‐points related to OAB symptoms in the BoNTA 100 U group than in the placebo group. The present results are comparable with those from two phase III trials carried out in North America and Europe, in which there were mean changes of −2.65 and −2.95 in the primary end‐point, the mean change in daily UI episodes from baseline at week 12 after treatment with BoNTA 100 U;^15^, ^16^ these are in accordance with a change of −3.42 in the present study. These improvements in OAB symptom end‐points translated into a positive impact of BoNTA on the PRO, in which statistically significant improvements in the KHQ domain and OABSS total scores, and a positive response on the TBS compared with the placebo, were seen. These results are therefore not only statistically significant, but are also clinically meaningful, showing that BoNTA treatment can ameliorate OAB symptoms.

The significant efficacy of BoNTA in OAB patients who have a high level of UI at baseline and in whom prior therapy using OAB medications has failed might be explained by its local delivery and the proposed dual mechanism of action, targeting both the afferent and efferent pathways. Specifically, BoNTA directly blocks efferent acetylcholine release and acetylcholine‐mediated detrusor contraction,^12^ while additionally, it might inhibit the release of other vesicle‐mediated neurotransmitters, such as substance P and adenosine triphosphate, and could moderate the overexpression of certain receptors responsible for inappropriate afferent signaling in OAB, such as P2X_3_ receptor and TRPV1, which might result in suppression of urgency.^13^, ^14^

With respect to safety, BoNTA 100 U was well tolerated in this study. AEs and treatment‐related AEs, those AEs that were higher in the BoNTA group than in the placebo group, were localized to the urinary tract, and included UTIs, dysuria, urinary retention and PVR urine volume increased. Although more patients in the BoNTA group experienced PVR urine volumes ≥100 mL, the proportion of patients who initiated CIC was just 6%. Most AEs that occurred in the BoNTA group were mild or moderate in severity, and the rate of study discontinuation was quite low. These results were similar to those in the trials carried out in North America and Europe.^15^, ^16^

One limitation of the present study is that the definition of UTI was based purely on laboratory results from urine culture and leukocyturia, and was not dependent on patients’ symptoms, which might be different in clinical practice. This could have led to a higher incidence of UTI (13%) than that seen in clinical practice. In one retrospective study, the incidence of UTI was lower than that in the present study.^25^

Regarding SAEs, the dysarthria that occurred in one patient in the BoNTA group was assessed as being related to the study drug, because the investigator could not rule out this possibility. However, the investigator suspected that it might have been related to the local anesthesia used concomitantly during BoNTA administration. The SAE occurred on the injection day and was resolved the next day, which seems to be inconsistent with the pharmacology of BoNTA.

In conclusion, treatment with BoNTA 100 U resulted in significant and clinically relevant improvements in OAB symptoms and PRO, and was well tolerated in Japanese patients with OAB and UI who were inadequately managed with OAB medications. These results suggest that BoNTA could be an important new treatment option for Japanese OAB patients who fail to respond to OAB medications, such as anticholinergics and/or β_3_‐adrenergic receptor agonists, due to poor response and/or tolerability.

## Conflict of interest

Osamu Yokoyama has received consultancy fees from Astellas, GlaxoSmithKline, Kissei, Kyorin, Nippon Shinyaku, Pfizer and Taiho; and grants from Astellas, Eisai, Kissei, Taiho and Takeda. Masashi Honda has received consultancy fees from GlaxoSmithKline. Tomonori Yamanishi has received grants from Astellas, Kyorin and Taiho. Yuki Sekiguchi has no conflict of interest. Kenji Fujii, Takashi Nakayama and Takao Mogi are employees of GlaxoSmithKline.

## Appendix

A complete list of names and affiliations of the principal investigators of the study:

Takeya Kitta, Hokkaido University Hospital; Chikara Ohyama, Hirosaki University School of Medicine and Hospital; Koichi Kambe, Senseki Hospital; Yoshikatsu Tanahashi, The Urology Office of Tanahashi; Isoji Sasagawa, Yamagata Tokushukai Hospital; Mikinobu Ootani, Ibaraki Prefectural Central Hospital; Eiyu Nozawa, Mito Red Cross Hospital; Tomonori Yamanishi, Dokkyo Medical University Hospital; Toshihiko Saito, Chiba Central Medical Center; Fumio Manabe, Touyu Clinic Shinmatsudo; Yasutomo Suzuki, Nippon Medical School Chiba Hokusoh Hospital; Hikaru Tomoe, Tokyo Women’s Medical University Medical Center East; Satoru Takahashi, Nihon University Itabashi Hospital; Nozomu Kawata, Nihon University Hospital; Hisashi Matsushima, Tokyo Metropolitan Police Hospital; Taketo Kawai, The University of Tokyo Hospital; Manami Kinjo, Kyorin University Hospital; Takashi Fukagai, Showa University Koto Toyosu Hospital; Atsuko Takahashi, Center Hospital of the National Center for Global Health and Medicine; Fumio Nakajima, Keiyu Hospital; Haruaki Sasaki, Showa University Fujigaoka Hospital; Norihiko Okuno, Sagamihara National Hospital; Yuki Sekiguchi, Medical Corporation LEADING GIRLS Women’s Clinic LUNA Next Stage; Toshiyuki Itoi, Niigata Rinko Hospital; Yoshiyuki Ishiura, Japan Organization of Occupational Health and Safety Toyama Rosai Hospital; Atsushi Mizokami, Kanazawa University Hospital; Yosuke Matsuta, University of Fukui Hospital; Norifumi Sawada, University of Yamanashi Hospital; Kosei Miwa, Japanese Red Cross Gifu Hospital; Atsushi Otsuka, Hamamatsu University School of Medicine, University Hospital; Yasuhito Funahashi, Nagoya University Hospital; Masaki Yoshida, National Center for Geriatrics and Gerontology; Motoi Tobiume, Japan Organization of Occupational Health and Safety, Asahi Rosai Hospital; Akihiro Kawauchi, Shiga University of Medical Science Hospital; Hiroshi Okuno, National Hospital Organization Kyoto Medical Center; Terukazu Nakamura, Saiseikai Suita Hospital; Noriko Ninomiya, Medical Corporation LEADING GIRLS Women’s Clinic LUNA Shinsaibashi; Chie Matsushita, Osaka Gyoumeikan Hospital; Atsushi Takenaka, Tottori University Hospital; Takeshi Watanabe, Watanabe Clinic; Toyohiko Watanabe, Okayama University Hospital; Noritaka Ishito, Kurashiki Medical Center/Kurashiki Medical Clinic; Yasuyo Yamamoto, Tokushima University Hospital; Hiroshi Ikeda, Kitakyushu General Hospital; Narihito Seki, Kyushu Central Hospital of the Mutual Aid Association of Public School Teachers; Shigehiro Kanegae, Keirindo Clinic; Kenichi Suyama, Medical Corporation Suyama Urology Clinic; Kazuyuki Sagiyama, Sagiyama Urology Clinic; Noriaki Tokuda, Saga‐Ken Medical Centre Koseikan; Hideki Sakai, Nagasaki University Hospital; Hiroaki Nishimura, Kagoshima University Hospital; Motoshi Kawahara, Kawahara Urological Clinic.
